# Immunodeficient patient experience of emergency switch from intravenous to rapid push subcutaneous immunoglobulin replacement therapy during coronavirus disease 2019 shielding

**DOI:** 10.1097/ACI.0000000000000864

**Published:** 2022-09-27

**Authors:** Clare Morgan, Stephen Jolles, Mark J. Ponsford, Kimberly Evans, Emily Carne

**Affiliations:** a School of Health and Social Care, Swansea University, Swansea, Wales; b Immunodeficiency Centre for Wales, University Hospital of Wales, Cardiff; c Division of Infection and Immunity, School of Medicine, Cardiff University, Cardiff, UK

**Keywords:** coronavirus disease 2019, immunoglobulin replacement therapy, intravenous immunoglobulin, patient reported outcome measures, rapid push subcutaneous immunoglobulin

## Abstract

Welsh immunodeficient patients on immunoglobulin replacement therapy (IgRT) who were considered high risk for severe coronavirus disease 2019 (COVID-19) were directed to shield. Consequently, patients receiving hospital-based intravenous immunoglobulin (IVIg) quickly transitioned to home-based self-administered subcutaneous immunoglobulin (SCIg). This evaluation aimed to assess patients’ perceptions and experiences and laboratory outcomes of emergency IgRT transition during COVID-19.

**Recent findings:**

A quick transition from in-hospital IVIg to home-based rapid push SCIg is achievable, however, patient IgRT administration preference remains key outside of emergency shielding measures.

**Summary:**

Subjective self-reported experiences (*n* = 23) and objective immunoglobulin G (IgG) concentration (*n* = 28) assessments were prospectively collected from patients pre/post-IgRT switch. In total, 41/55 (75%) patients transitioned from IVIg to rapid push SCIg and all completed training to self-administer subcutaneously within 24 days. Twenty-two percent (*n* = 5) of patients preferred SCIg and 35% (*n* = 8) wanted to return to hospital-based IVIg at 6 weeks post-transition. Mean IgG levels were similar pre vs. post-SCIg switch (10.3 g/l vs. 10.6 g/l, respectively). Patients reported greater infection anxiety during COVID-19 and adapted behaviours to mitigate risk. Although a third of patients wished to return to IVIg following cessation of shielding, over time the percentage electing to remain on SCIg rose from 22% to 59%.

## INTRODUCTION

Symptoms of coronavirus disease 2019 (COVID-19) range from asymptomatic infection to mild disease (persistent cough, fever, and fatigue) with the most clinically vulnerable patients experiencing more severe respiratory involvement, with around 5% having critical disease such as sepsis and multi organ failure [1,2]. On 16 March 2020, the UK government advised that all vulnerable, at-risk patients should shield themselves at home for 12 weeks [3–5]. Patients with immunodeficiency are at known increased risk of developing recurrent severe non-COVID-19 infections, with antibody deficient patients receiving life-long immunoglobulin replacement therapy (IgRT) to mitigate infection risk [6^▪^], though IgRT does not provide protection from novel pathogens. The risk of both severe and persistent COVID-19 infection is higher in immunocompromised patients [7–9,10^▪^]. Consequently, immunodeficient patients were identified as being clinically extremely vulnerable (CEV) to developing serious illness as a result of COVID-19 and asked to shield.

At this time, the Immunodeficiency Centre for Wales (ICW) cared for 226 adult patients with primary (PID), secondary immunodeficiency (SID) or predominant antibody deficiency receiving IgRT. IgRT is administered intravenously (IVIg) or subcutaneously (SCIg), both of which are effective and well tolerated treatments [6^▪^,^11^]. Patient choice is advocated to minimize the treatment burden associated with lifelong IgRT [12,13], thus increasing the likelihood of continued treatment compliance and ensuring protection against infections [14]. Patients and their physicians consider multiple factors when selecting IgRT regimens including: frequency and route of administration, device used to administer immunoglobulin G (IgG), IgG infusion volume, number of needle-sticks/infusion sites, overall needle burden and venous access, patient lifestyle, the occurrence and management of adverse events (AEs) and education and training required [6^▪^].

IVIg is typically administered at regular outpatient infusion suites at 3 or 4 weekly intervals [6^▪^]. Frequent hospital attendance was considered a breach of shielding and a risk of nosocomial exposure to COVID-19 [15,16]. Therefore, patients receiving hospital-based IgRT were assessed and where possible offered emergency transition to home-based SCIg. When this was not possible, home-based IVIg administered via Immunology Clinical Nurse Specialists was undertaken. Rapid push SCIg can be administered via a needle and syringe using smaller and more frequent doses (taking approximately 5–20 min) than traditional weekly infusion pump administered SCIg [17,18^▪^]. Due to its relative simplicity, transition to rapid push SCIg was selected as the most efficient manner to support rapid shielding whilst ensuring continued access to IgRT.

At the ICW, 53 of 226 patients had previously chosen to receive hospital-based IVIg, 171 home-based SCIg, and two patients were newly diagnosed and had not yet commenced any form of IgRT (IgRT-naïve). Given this unique emergency situation and recognizing this population encompasses 53 patients who had previously declined SCIg, together with a significantly shortened SCIg training period, we undertook a service evaluation to explore patients’ perceptions and self-reported experiences of their emergency IgRT transition which involved the alteration of their chosen route for IgRT. We also assessed IgG trough levels before and after transition from IVIg to SCIg. 

**Box 1:**
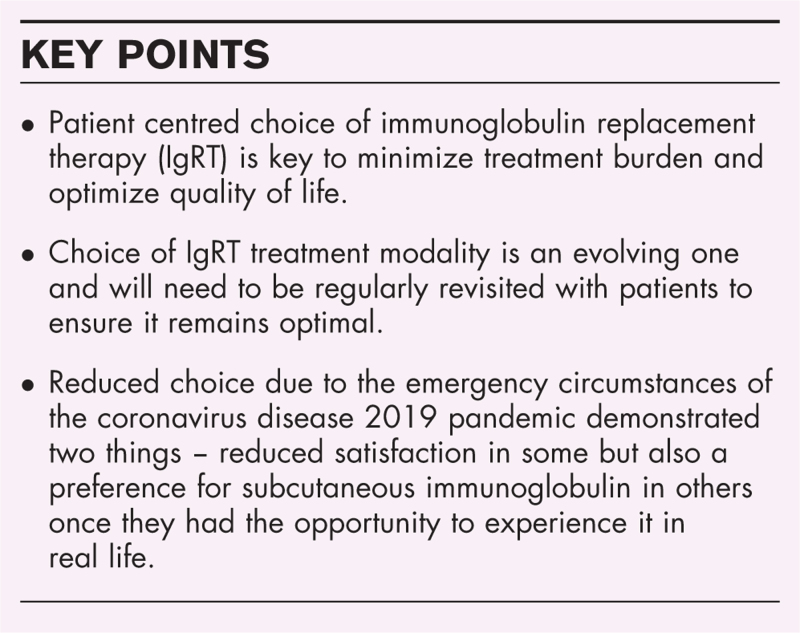
no caption available

## METHODS

### Service evaluation overview

This work was undertaken as a service evaluation according to the definitions of the UK Policy Framework for Health and Social Care Research and was therefore exempt from ethical board approval.

### Patient inclusion

Patients with immunodeficiency who were either treatment naïve or being treated with IVIg under ICW and had actively chosen IVIg over home administration of SCIg were invited to attend a one-off hospital training session to self-administer rapid push SCIg at home. Training sessions were undertaken by Immunology Clinical Nurse Specialists. Patients who had previously administered SCIg independently at some point had already completed the routine six-week training course. Those who did not require accelerated training were switched to home-based SCIg immediately.

### Immunoglobulin replacement therapy doses

SCIg doses were calculated based on a 1:1 dose transition to ensure patients received an equivalent dose of weekly SCIg corresponding to their previous 3 of 4 weekly IVIg regime. Wales Immunoglobulin Strategy Group (WISG) and the Welsh Blood Service coordinated SCIg supply to ensure patient access for the emergency switch.

### Data collection

Patient-reported assessments and laboratory assessments were prospectively collected before and after the IgRT transition. Completion of the self-reported questionnaires was voluntary, with telephone contact by a Clinical Nurse Specialist during routine follow-up. Patients were given up to 8t weeks to respond post-SCIg transition. Clinical and laboratory records/data prior to and following the IgRT route switch were evaluated.

### Questionnaires

Patient-reported treatment satisfaction of the IgRT transition from hospital-based IVIg to home-based rapid push SCIg was assessed using the validated Treatment Satisfaction Questionnaire for Medication Version II (TSQM II) [19]. The TSQM II evaluates the effectiveness of treatment, adverse effects/AEs, convenience of use, and overall global satisfaction with a maximum score of 100 for each component. A higher score corresponds to greater satisfaction, fewer AEs and is associated with better treatment adherence [19]. Patient satisfaction was assessed between 5 and 8 weeks after patients had started SCIg self-administration.

The patient experience questionnaire considered: reasons for previously declining home-based SCIg, satisfaction with the rapid push SCIg training, current plans to remain on SCIg or return to hospital-based IVIg and anxiety/concern over respiratory infections before and during the COVID-19 pandemic. A score of 100% indicates that the patient was extremely satisfied and a score of 0% indicates that the patient was extremely unsatisfied.

### Immunoglobulin G levels

During routine monitoring, IgG trough levels were obtained approximately every 3 months at patients’ regular IVIg hospital infusions, and every 3 months after starting home-based SCIg. Mean IgG levels were calculated before the SCIg switch (September 2019 to March 2020) and every 3 months after the switch, if available (March 2020 to December 2020). All testing was performed in the UK Accreditation Service accredited Immunology Laboratory at the University Hospital of Wales.

### Statistical analysis

To compare changes pre and post-SCIg switch, statistical analyses were performed. For TSQM II scores a paired *t*-test was used. For IgG trough levels, changes at a patient and group level were assessed. At the patient level, a paired *t*-test was used (only patients contributing data at both time points were included in the analysis). The mean value was used when there were multiple measurements per patient in any one time (pre or post-SCIg switch). At the group level, the mean value of all the individual measurements across the patient cohort was assessed. Group analysis was performed using linear two-level (mixed) regression models with individual measurements nested within patients. Data was curated in Microsoft Excel and analysis performed using Stata (version 15.1).

## RESULTS

### Patient identification and characteristics

**FIGURE 1:**
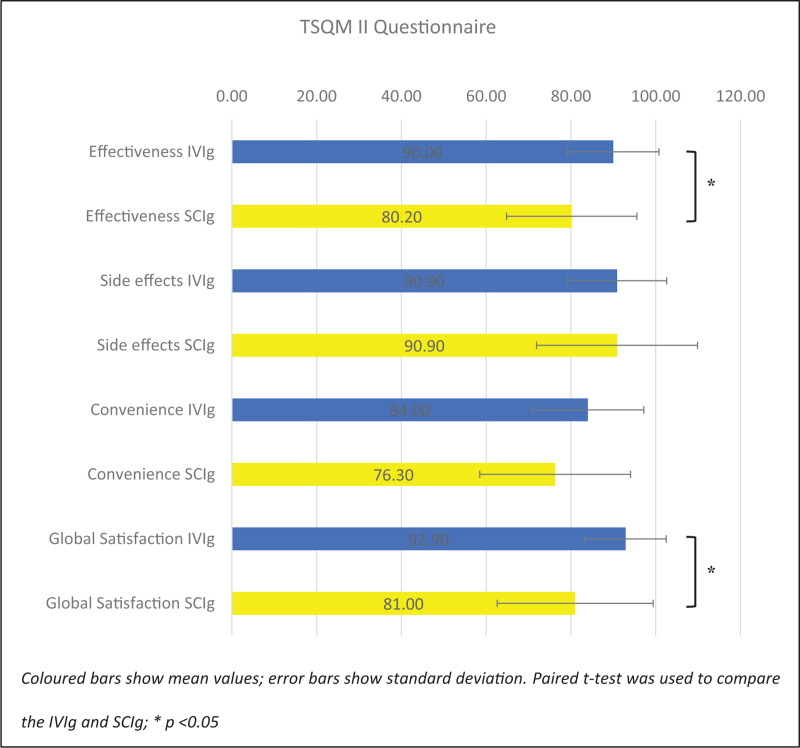
TSQM II medication satisfaction scores for patients switching from IVIg to SCIg (*n* = 21). IVIg, intravenous immunoglobulin; SCIg, subcutaneous immunoglobulin; TSQM II, Treatment Satisfaction Questionnaire for Medication Version II.

Within seven days of the shielding announcement, 55 patients were contacted to discuss the rationale to transition from IVIg to SCIg. We identified 53 patients receiving regular IVIg infusions and two newly diagnosed IgRT-naïve patients (Figure 1, Supplemental Digital Content). In total, 41 accepted to transition; 31 patients were SCIg naïve (including two IgRT-naïve patients), 10 patients had received SCIg prior to their current IVIg infusions; eight of whom required further training on administering SCIg, whilst 2 patients remained confident and required no further training (Figure 1, Supplemental Digital Content). The remaining 14 patients were unable to switch to SCIg for clinical reasons including dexterity, frailty or inability to perform SCIg infusions safely. These 14 patients were excluded from the switch survey and remained on IVIg administered at home (*n* = 13) or hospital (*n* = 1) by Immunology Clinical Nurse Specialists (CNS). No patient opted to discontinue IgRT. Baseline patient characteristics (*n* = 41) are shown in Table 1.

**Table 1:** Baseline characteristics of survey participants (*n* = 41)

Characteristic	Patients who switched from IVIg to SCIg (*n* = 41)
Sex, *n* (%)	
Female	26 (63)
Male	15 (37)
Age (years)	
Median (range)	58 (19–88)
Classification of immunodeficiency, *n* (%)	
Common variable immunodeficiency	16 (39)
Probable common variable immunodeficiency	1 (2)
Severe combined immunodeficiency	1 (2)
Hypogammaglobulinaemia	6 (15)
Secondary hypogammaglobulinemia	3 (7)
X-linked agammaglobulinaemia	1 (2)
Panhypogammaglobulinaemia	2 (5)
Secondary panhypogammaglobulinaemia	2 (5)
Specific antibody deficiency	3 (7)
Probable specific antibody deficiency	1 (2)
Secondary antibody deficiency	2 (5)
Low IgM	1 (2)
Myeloma	1 (2)
MGUS – IgG kappa protein, low IgM	1 (2)
Relevant co-morbidities/treatments, *n* (%)	
Immune thrombocytopenia	2 (5)
Bronchiectasis	12 (29)
Lymphoma	2 (5)
Chronic lymphocytic leukaemia	1 (2)
COPD	1 (2)
Neutropenia	1 (2)
Previous smoker	6 (15)
Rituximab use	4 (10)
N/A	17 (41)
Previous IgG therapy, *n* (%)	
Naïve	2 (5)
IVIg 2 weekly	4 (10)
IVIg 3 weekly	32 (78)
SCIg 2 weekly	1 (2)
SCIg 3 weekly	2 (5)

COPD, chronic obstructive pulmonary disease; Ig, immunoglobulin; IVIg, intravenous immunoglobulin; MGUS, monoclonal gammopathy of unknown significance; SCIg, subcutaneous immunoglobulin.

### Time to subcutaneous immunoglobulin training (rollout of home immunoglobulin replacement therapy)

**FIGURE 2:**
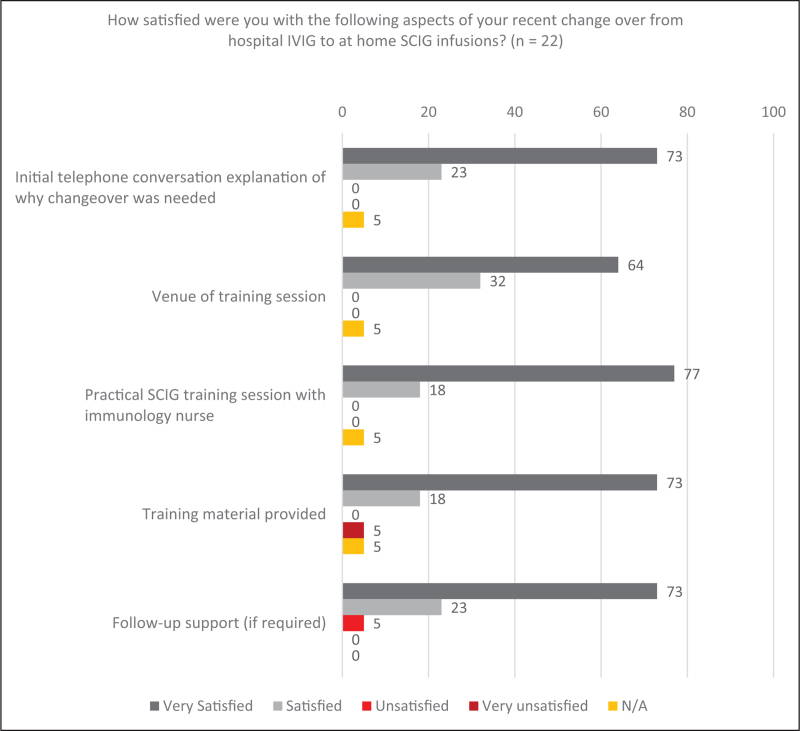
Patient experience of IVIg to SCIg switch. IVIg, intravenous immunoglobulin; SCIg, subcutaneous immunoglobulin.

Within 14 days of shielding, 71% (*n* = 29) of eligible patients (1 IgRT-naïve, and 28 IVIg-experienced patients) had been trained to administer SCIg at home. By day 21, a further 22% (*n* = 9) of patients (one IgRT-naïve and eight IVIg-experienced) had completed training. By day 24, one additional patient had completed training. The remaining two patients had previously received SCIg training and were considered competent to return to home-based administration without additional training (Figure 2).

### Treatment satisfaction

Out of 41 patients, 22 (53%) returned responses. One patient had never received IVIg before and was excluded from comparative analysis. Overall, greater satisfaction scores were observed with prepandemic IVIg compared to current SCIg (Fig. 1), with mean TSQM II scores significantly higher for IVIg than SCIg in effectiveness (90.0 vs. 80.2; *P* = 0.03) and global satisfaction (92.9 vs. 81.0; *P* = 0.01). Convenience of IVIg was also greater but did not significantly differ compared with SCIg (84.0 vs. 76.3; *P* = 0.06). For adverse effects, mean TSQM II scores were comparable (90.9 vs. 90.9; *P* = 1.00).

### Patient experience

Of the 41 patients, 23 (56%) provided their responses to the patient experience questionnaire. As patient-reported completion rates varied for the questionnaire we have indicated the number of respondents in Table 1, Supplemental Digital Content and Table 2, where applicable.

**Table 2:** Patients’ experience with rapid push SCIg

How satisfied are you with your SCIg infusions at home since March 2020 (0% = extremely unsatisfied, 100% = extremely satisfied) (*n* = 22)		
		*n* (%)
	0–9%	2 (9)
	40–49%	1 (5)
	50–59%	3 (14)
	60–69%	1 (5)
	70–79%	3 (14)
	80–89%	2 (9)
	90–99%	6 (27)
	100%	4 (18)

Would you like to remain on SCIg infusions at home once the current government lockdown due to coronavirus is over? (*n* = 23)		
		*n* (%)
	No, I want to return to IVIg in hospital as soon as possible	8 (35)
	Maybe, I have not made my mind up yet	10 (43)
	Yes, I now prefer administering SCIg at home	5 (22)

IVIg, intravenous immunoglobulin; SCIg, subcutaneous immunoglobulin.

#### Intravenous immunoglobulin infusions

Prior to March 2020, 68% (*n* = 15/22) of patients had been treated with IVIg for three or more years, and 32% (*n* = 7/22) treated for over 10 years. Reasons patients gave for choosing IVIg encompassed not wanting to self-administer injections at home, preference for hospital-based IgRT and less frequent infusions required (Table 1, Supplemental Digital Content). In total, 18% (*n* = 4/22) of patients reported that they had never been offered SCIg at home before (including the two treatment-naïve patients) potentially as they had transferred from Haematology where SCIg is not routinely available. Overall, patients expressed high levels of satisfaction with IVIg, with 91% (*n* = 20/22) reporting satisfaction scores between 90–100% (extremely satisfied). Several reasons given for these high satisfaction scores included their IVIg infusion schedules and interactions with health professionals.

#### Training

Overall, patients were very satisfied with their treatment transition, with 77% (*n* = 17/22) of patients very satisfied with the practical SCIg training session and 73% (*n* = 16/22) very satisfied with the training material provided (Fig. 2). Patients were also very satisfied with the follow up support (73%; *n* = 16/22) and training venue (64%; *n* = 14/22). Fewer than 5% of patients stated they were either unsatisfied or less than satisfied with their treatment transition. Responses given for their dissatisfaction identified temporary issues in homecare delivery/supplies of SCIg and ancillaries, and a preference for face-to-face contact rather than remote support post-SCIg training.

#### Subcutaneous immunoglobulin infusions

After the IgRT transition, 45% (*n* = 10/22) of patients reported satisfaction levels between 90% and 100% (extremely satisfied) for their SCIg infusions and only 9% (*n* = 2/22) reported levels between 0 and 9% (extremely unsatisfied) (Table 2). Patients cited improved convenience and independence of at-home self-administration, ease-of-use and efficient training for their high satisfaction with SCIg. In patients who were not satisfied with their SCIg infusions, several reasons were reported. First, treatment adverse effects, with some patients requiring higher doses of SCIg and experiencing significant pain and swelling at the infusion site. Second, apprehension over self-administration and lack of support from hospital staff. Finally, patients cited reduced freedom of treatment choice due to the pandemic.

At 6 weeks post-transition, approximately a third of questionnaire respondents (35%; *n* = 8/23) wanted to return to hospital-based IVIg as soon as possible whilst 22% (*n* = 5/23) preferred SCIg and wished to remain on this long term (Table 2). Patients who preferred SCIg highlighted treatment flexibility, convenience and the short duration of infusions. The remaining 43% (*n* = 10/23) were undecided on how they wished to administer their IgRT long term. Over time, more patients (59%; *n* = 22/37) have chosen to remain on SCIg, with 38% (*n* = 14/37) returned to their previous hospital IVIg infusions. Three percent (*n* = 1) patients are receiving SCIg infusions in hospital. Ten percent (*n* = 4) patients have died since questionnaires were returned.

### Anxiety pre- and post-coronavirus disease 2019

Overall, a greater percentage of immunodeficient patients were severely anxious about catching a respiratory infection after March 2020 (59%) compared with December 2019 (14%) (Figure 3A, Supplemental Digital Content). This is a notably higher than the 30% prevalence of probable anxiety in United Kingdom residents during the COVID-19 pandemic, identified via longitudinal evidence from the European COVID Survey [20], with these anxiety levels positively correlated to income difficulties and lower health-related quality of life. Similar changes in patient anxiety after March 2020 were seen in relation to catching an infection when attending hospital appointments (Figure 3B, Supplemental Digital Content) and when carrying out normal daily activities (Figure 3C, Supplemental Digital Content). Behaviour was modified (Figure 4, Supplemental Digital Content) with avoidance of those with a cough/fever/unwell, public transport, public places alongside greater home isolation and reduced social contact.

### Immunoglobulin G levels

Mean IgG trough levels pre and post-SCIg switch were available in 28 of the 41 patients (Figure 5, Supplemental Digital Content). In the remaining 13 patients, IgG levels were not analysed or included in the comparison (some patients had reverted back to IVIg before post-switch blood samples were collected and others were treatment naïve). At a group level, no significant difference in IgG trough levels were observed after IgRT transition, with a group mean IgG level of 10.3 g/l measured pre-SCIg switch compared to a group mean IgG level of 10.6 g/l measured post-SCIg switch (*P* = 0.15). At an individual patient level, no significant difference was observed in the IgG trough values pre and post-SCIg switch (*P* = 0.25). However, in eight of these patients, lower IgG trough levels were observed after the SCIg switch, with differences ranging from 0.02 to 3.93 g/l lower. Routine, ongoing IgG monitoring is standard practice to ensure individual dose optimization following any change in a patient's IgG infusion regime. Of these eight patients, over time, two returned to their previous IVIg administration as soon as they were able to, five have remained on their transitioned SCIg dose, and one patient required an increase in weekly SCIg dose.

## CONCLUSION

The unique aspect of this evaluation is that it provides insight into patient perspectives and treatment satisfaction when choice and clinical support in IgRT administration is reduced by external emergency circumstances. Understanding these factors is key for therapy planning and management, as therapy choice can influence patient wellbeing and quality of life. The need for managing patient perceptions and expectations of SCIg, and facilitating a return to previously preferred administration routes if desired post emergency is highlighted.

Previous work has shown improvements in treatment satisfaction and patient quality of life when patients have transitioned from hospital-based IVIg to home-based SCIg [21–25]. Given the unprecedented pace and nature of the emergency transition, we evaluated patient treatment satisfaction with IVIg or SCIg using the TSQM II questionnaire. Greater satisfaction scores were observed across three of the four domains for IVIg compared with rapid push SCIg (*convenience*, *effectiveness*, and *global satisfaction*, with the latter two showing significant differences). Results from the patient experience questionnaire, completed up to six weeks post IVIg to SCIg transition reported that approximately a third of patients wished to return to IVIg as soon as possible, with 43% undecided about transitioning to SCIg permanently. However, it is important to interpret these findings in the context of a cohort already established on IVIg, and where many had previously chosen IVIg over SCIg (a third of patients participating in the survey had been on IVIg in hospital for >10 years). Whilst patient preference is carefully considered at the ICW, patient choice was significantly diminished during COVID-19 due to the emergency clinical need to shield. Questionnaire responses and this lack of patient choice is reflected in the significant number of patients who over time have since returned to their previous IVIg administration (38%; *n* = 14/37).

Initially, poor SCIg treatment satisfaction scores may have been observed for several reasons including local adverse effects (which often diminish over time), a perceived lack of treatment choice, significantly shortened SCIg training and lack of ongoing face-to-face support and lack of treatment familiarity. This reinforces the benefits of longer, in depth standards of pre-emergency SCIg training and ongoing in-person clinical support once emergency restrictions are no longer required.

Although the questionnaire findings contrast with prior studies, 22% of patients had chosen to continue with SCIg within 6 weeks of transitioning, highlighting improved treatment flexibility and convenience with home self-administration and shorter infusion times. Interestingly, this proportion of patients wished to remain on SCIg, despite previously declining this administration route. Over time, the majority of the overall transitioned cohort (59%; *n* = 22/37) have remained on SCIg, indicating these individuals have likely become increasingly confident and satisfied with SCIg home administration but also reflecting potential ongoing fear of nosocomial infection on returning to in hospital IVIg. This highlights that there are still barriers to IgRT administration awareness and optimization in a significant number of patients, perhaps related to a perceived inertia to change when established on a treatment regimen and significantly different outcomes when choices are made based personal experience vs. ‘on paper’ discussions. These findings demonstrate that IgRT administration route should be part of a process of continuous review, with ongoing discussions and potentially consideration of a trial of a different route taking place between medical, nursing staff, and patients. The duration of such a trial will need to be of sufficient duration to allow patients to fully evaluate the new route of treatment and overcome initial concerns as was evident from the increasing numbers of patients remaining on SCIg over time. Furthermore, the importance of evolving individual treatment needs, and patient preference/choice and how best to inform that choice should remain at the core of treatment decision making.

Our routine practice for SCIg training includes a minimum of four to six face-to-face training sessions, followed by a home assessment for the first infusion. However, due to the pandemic, training was reduced to just one hospital session. Considering the emergency context of COVID-19, patients reported a high degree of satisfaction across all aspects of SCIg training. A minority of patients (5%) reported a lack of satisfaction with their SCIg training and cited reasons relating to the training material provided, and lack of ongoing face-to-face support. Additionally, patients commented on the support, professionalism and interactions with care staff as reasons for their previously high satisfaction with IVIg treatment. This highlight the importance of staff support and interaction for treatment satisfaction and was especially evident in patients, largely of the settled view that they preferred IVIg, and who stated the reduced personal choice and nursing support imposed by the pandemic impacted their treatment satisfaction.

This study also demonstrates that a rapid transition from IVIg to rapid push SCIg is feasible and while overall IgG trough levels remained stable following a 1:1 switch further individual dose adjustment will be required in some. A rapid emergency switch of this nature is not possible without sufficient supply of SCIg products, in this case coordinated by Welsh Blood Service and Wales Immunoglobulin Strategy Group. The lower IgG trough levels observed in some may also be influenced by an individual's reluctance to use SCIg due to their perception or reported AEs and impaired treatment adherence. Given that in general SCIg AEs decrease with time, the comparison of recently commenced SCIg with stable IVIg may bias toward improved perception of IVIg therapy [26,27]. Furthermore, patients with a needle phobia may be less compliant, resulting in reduced IgG administration. Although telephone support with a Clinical Nurse Specialist was available to all patients post SCIg transition, additional face-to-face support from the standard, longer training programme may have been beneficial [25].

## Acknowledgements

*Alison Pritchard, Frances McGuire, Anthony Matthews, and Michelle Norton contributed to data collection and editorial assistance was provided Meridian HealthComms Ltd, funded by CSL Behring.*

### Financial support and sponsorship

*M.J.P. is supported by the Welsh Clinical Academic Training (WCAT) programme and a Career Development Award from the Association of Clinical Pathologists and is a participant in the NIH Graduate Partnership Programme.*

### Conflicts of interest

*S.J. has received support for conferences, speaker, advisory boards, trials, data and safety monitoring boards, and projects with CSL Behring, Takeda, Swedish Orphan Biovitrum, Biotest, Binding Site, Grifols, BPL, Octapharma, LFB, Pharming, GSK, Weatherden, Zarodex, Sanofi, and UCB Pharma.*

## Supplementary Material

Supplemental Digital Content

## Supplementary Material

Supplemental Digital Content

## Supplementary Material

Supplemental Digital Content

## Supplementary Material

Supplemental Digital Content

## Supplementary Material

Supplemental Digital Content

## Supplementary Material

Supplemental Digital Content

## REFERENCES AND RECOMMENDED READING

Papers of particular interest, published within the annual period of review, have been highlighted as:

▪ of special interest
▪▪ of outstanding interest

## References

[1] Atzrodt CL Maknojia I McCarthy RDP. A guide to COVID-19: a global pandemic caused by the novel coronavirus SARS-CoV-2. FEBS J 2020; 287:3633–3650. 32446285 10.1111/febs.15375 PMC7283703

[2] Wu Z McGoogan JM. Characteristics of and important lessons from the coronavirus disease 2019 (COVID-19) outbreak in China: summary of a report of 72 314 cases from the Chinese Center for Disease Control and Prevention. JAMA 2020; 323:1239–1242. 32091533 10.1001/jama.2020.2648

[3] Public Health England. Guidance on shielding and protecting people who are clinically extremely vulnerable from COVID-19. 2020. Available at: https://www.gov.uk/government/publications/guidance-on-shielding-and-protecting-extremely-vulnerable-persons-from-covid-19/guidance-on-shielding-and-protecting-extremely-vulnerable-persons-from-covid-19.

[4] Welsh Government. Guidance on protecting people defined on medical grounds as clinically extremely vulnerable from coronavirus (COVID-19) – previously known as ‘shielding’. 2020. Available at: https://gov.wales/guidance-on-shielding-and-protecting-people-defined-on-medical-grounds-as-extremely-vulnerable-from-coronavirus-covid-19-html.

[5] Freedman L. Strategy for a pandemic: the UK and COVID-19. Survival 2020; 62:25–76.

[6▪] Jolles S Orange JS Gardulf A. Current treatment options with immunoglobulin G for the individualization of care in patients with primary immunodeficiency disease. Clin Exp Immunol 2015; 179:146–160. 25384609 10.1111/cei.12485 PMC4298393

[7] Moran E Cook T Goodman AL. Persistent SARS-CoV-2 infection: the urgent need for access to treatment and trials. Lancet Infect Dis 2021; 21:1345–1347. 34411531 10.1016/S1473-3099(21)00464-3 PMC8367192

[8] Bradley RE Ponsford MJ Scurr MJ. Persistent COVID-19 Infection in Wiskott-Aldrich Syndrome Cleared Following Therapeutic Vaccination: a case report. J Clin Immunol 2021; 42:32–35. 34714497 10.1007/s10875-021-01158-5 PMC8554737

[9] Brown LK Moran E Goodman A. Treatment of chronic or relapsing COVID-19 in immunodeficiency. J Allergy Clin Immunol 2022; 149:557–561. 34780850 10.1016/j.jaci.2021.10.031 PMC8585958

[10▪] Shields AM Anantharachagan A Arumugakani G. Outcomes following SARS-CoV-2 infection in patients with primary and secondary immunodeficiency in the UK. Clin Exp Immunol 2022; uxac008. [Online ahead of print]. 10.1093/cei/uxac008 PMC8807296 35641155

[11] Berger M. Principles of and advances in immunoglobulin replacement therapy for primary immunodeficiency. Immunol Allergy Clin North Am 2008; 28:413–437. 18424340 10.1016/j.iac.2008.01.008 PMC7127239

[12] Jiang F Torgerson TR Ayars AG. Health-related quality of life in patients with primary immunodeficiency disease. Allergy Asthma Clin Immunol 2015; 11:27. 26421019 10.1186/s13223-015-0092-y PMC4587876

[13] Grigoriadou S Clubbe R Garcez T. British Society for Immunology & United Kingdom Primary Immunodeficiency Network (UKPIN) consensus guideline for the management of immunoglobulin replacement therapy. Clin Exp Immunol 2022; uxac070. doi: 10.1093/cei/uxac070. Online ahead of print. 10.1093/cei/uxac070 PMC9585546 35924867

[14] Nicolay U Haag S Eichmann F. Measuring treatment satisfaction in patients with primary immunodeficiency diseases receiving lifelong immunoglobulin replacement therapy. Qual Life Res 2005; 14:1683–1691. 16119180 10.1007/s11136-005-1746-x

[15] Ponsford MJ Ward TJC Stoneham SM. A systematic review and meta-analysis of inpatient mortality associated with nosocomial and community COVID-19 exposes the vulnerability of immunosuppressed adults. Front Immunol 2021; 12:744696. 34691049 10.3389/fimmu.2021.744696 PMC8526940

[16] Ponsford MJ Jefferies R Davies C. Burden of nosocomial COVID-19 in Wales: results from a multicentre retrospective observational study of 2508 hospitalised adults. Thorax 2021; 76:1246–1249. 34301738 10.1136/thoraxjnl-2021-216964 PMC8606436

[17] Shapiro RS. Subcutaneous immunoglobulin therapy given by subcutaneous rapid push vs infusion pump: a retrospective analysis. Ann Allergy Asthma Immunol 2013; 111:51–55. 23806460 10.1016/j.anai.2013.04.015

[18▪] Warnatz K Jolles S Agostini C. Subcutaneous Gammanorm(R) by pump or rapid push infusion: Impact of the device on quality of life in adult patients with primary immunodeficiencies. Clin Immunol 2022; 236:108938. 35121105 10.1016/j.clim.2022.108938

[19] Atkinson MJ Kumar R Cappelleri JC Hass SL. Hierarchical construct validity of the treatment satisfaction questionnaire for medication (TSQM version II) among outpatient pharmacy consumers. Value Health 2005; 8: (Suppl 1): S9–S24. 16336491 10.1111/j.1524-4733.2005.00066.x

[20] Hajek A Sabat I Neumann-Bohme S. Prevalence and determinants of probable depression and anxiety during the COVID-19 pandemic in seven countries: longitudinal evidence from the European COvid Survey (ECOS). J Affect Disord 2022; 299:517–524. 34920039 10.1016/j.jad.2021.12.029 PMC8684990

[21] Mallick R Jolles S Kanegane H. Treatment satisfaction with subcutaneous immunoglobulin replacement therapy in patients with primary immunodeficiency: a pooled analysis of six Hizentra® studies. J Clin Immunol 2018; 38:886–897. 30465179 10.1007/s10875-018-0562-3 PMC6292975

[22] Gardulf A Borte M Ochs HD Nicolay U. Prognostic factors for health-related quality of life in adults and children with primary antibody deficiencies receiving SCIG home therapy. Clin Immunol 2008; 126:81–88. 17964220 10.1016/j.clim.2007.06.009

[23] Gardulf A Nicolay U Math D. Children and adults with primary antibody deficiencies gain quality of life by subcutaneous IgG self-infusions at home. J Allergy Clin Immunol 2004; 114:936–942. 15480339 10.1016/j.jaci.2004.06.053

[24] Nicolay U Kiessling P Berger M. Health-related quality of life and treatment satisfaction in North American patients with primary immunodeficiency diseases receiving subcutaneous IgG self-infusions at home. J Clin Immunol 2006; 26:65–72. 16418804 10.1007/s10875-006-8905-x

[25] Ponsford M Carne E Kingdon C. Facilitated subcutaneous immunoglobulin (fSCIg) therapy – practical considerations. Clin Exp Immunol 2015; 182:302–313. 26288095 10.1111/cei.12694 PMC4636892

[26] Gardulf A Nicolay U Asensio O. Rapid subcutaneous IgG replacement therapy is effective and safe in children and adults with primary immunodeficiencies—a prospective, multinational study. J Clin Immunol 2006; 26:177–185. 16758340 10.1007/s10875-006-9002-x

[27] Jolles S Sleasman JW. Subcutaneous immunoglobulin replacement therapy with Hizentra, the first 20% SCIG preparation: a practical approach. Adv Ther 2011; 28:521–533. 21681653 10.1007/s12325-011-0036-y

