# Supplementary material for: Immunodeficient patient experience of emergency switch from intravenous to rapid push subcutaneous immunoglobulin replacement therapy during coronavirus disease 2019 shielding
Source: Curr Opin Allergy Clin Immunol. 2022 Sep 27;22(6):371–9. doi: 10.1097/ACI.0000000000000864 (PMC9612677; doi:10.1097/ACI.0000000000000864)
Supplement: Supplemental Digital Content [file coaci-22-371-s001.docx]

Supplementary Figure 1. Patient inclusion and exclusion based on prior immunoglobulin replacement therapy.


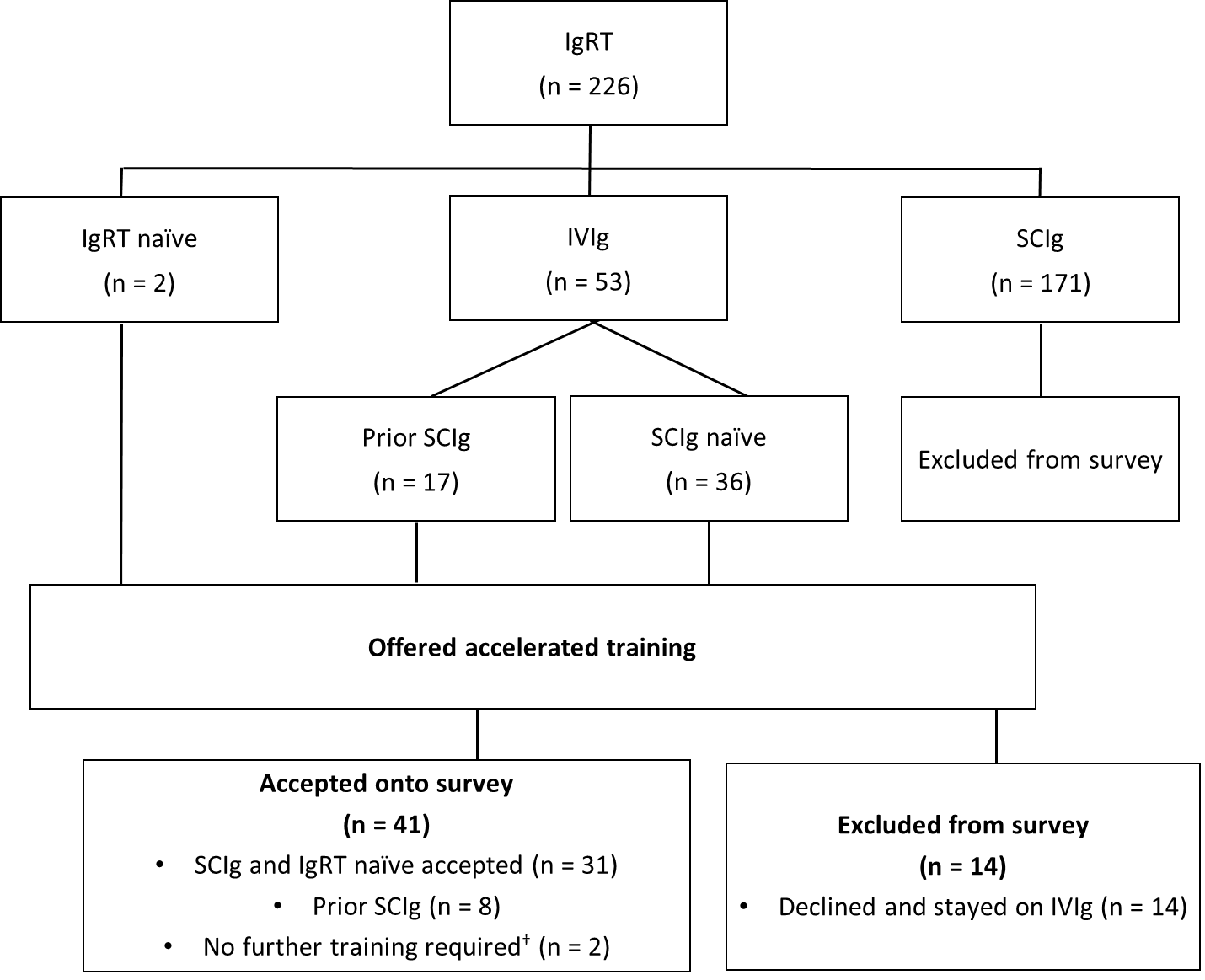


†*Patients had previously received SCIg training but reverted to IVIg.*

*Ig, immunoglobulin; IgRT, immunoglobulin replacement therapy; IVIg, intravenous immunoglobulin; SCIg, subcutaneous immunoglobulin*
