# Supplementary material for: Immunodeficient patient experience of emergency switch from intravenous to rapid push subcutaneous immunoglobulin replacement therapy during coronavirus disease 2019 shielding
Source: Curr Opin Allergy Clin Immunol. 2022 Sep 27;22(6):371–9. doi: 10.1097/ACI.0000000000000864 (PMC9612677; doi:10.1097/ACI.0000000000000864)
Supplement: Supplemental Digital Content [file coaci-22-371-s002.docx]

Figure 2: Time to SCIg training (n = 41).

*Day 0 refers to the 16th of March 2020 and the instruction for shielding of vulnerable patients by the U.K. government.*

*Ig, immunoglobulin; IVIg, intravenous immunoglobulin; SCIg, subcutaneous immunoglobulin.*
