# Supplementary material for: Immunodeficient patient experience of emergency switch from intravenous to rapid push subcutaneous immunoglobulin replacement therapy during coronavirus disease 2019 shielding
Source: Curr Opin Allergy Clin Immunol. 2022 Sep 27;22(6):371–9. doi: 10.1097/ACI.0000000000000864 (PMC9612677; doi:10.1097/ACI.0000000000000864)
Supplement: Supplemental Digital Content [file coaci-22-371-s003.docx]

Supplementary Figure 3: Patient anxiety scores pre- (before December 2019) and post- COVID-19 (after March 2020) regarding (A) catching a respiratory infection (n=21 and n=22, respectively), (B) catching an infection at the hospital (n=20 and n=19, respectively), and (C) catching a respiratory infection during daily activities (n=21 and n=19, respectively).

**A**

**B**

**C**
