# Supplementary material for: Immunodeficient patient experience of emergency switch from intravenous to rapid push subcutaneous immunoglobulin replacement therapy during coronavirus disease 2019 shielding
Source: Curr Opin Allergy Clin Immunol. 2022 Sep 27;22(6):371–9. doi: 10.1097/ACI.0000000000000864 (PMC9612677; doi:10.1097/ACI.0000000000000864)
Supplement: Supplemental Digital Content [file coaci-22-371-s004.docx]

Supplementary Figure 4. Patient behaviour pre- COVID-19 (before March 2020) and during -COVID-19 (after March 2020).
