# Supplementary material for: Immunodeficient patient experience of emergency switch from intravenous to rapid push subcutaneous immunoglobulin replacement therapy during coronavirus disease 2019 shielding
Source: Curr Opin Allergy Clin Immunol. 2022 Sep 27;22(6):371–9. doi: 10.1097/ACI.0000000000000864 (PMC9612677; doi:10.1097/ACI.0000000000000864)
Supplement: Supplemental Digital Content [file coaci-22-371-s005.docx]

Supplementary Figure 5: Mean individual patient Ig levels before and after SCIg switch (n = 28).

*Coloured bars show mean values; error bars show standard deviation.* *Paired t-test was used to compare individual patient Ig levels pre- and post-switch and linear multilevel (mixed) regression models were used to compare the group mean values pre- and post-SCIg switch.*

*Ig, immunoglobulin; SCIg, subcutaneous immunoglobulin.*
