# Supplementary material for: Immunodeficient patient experience of emergency switch from intravenous to rapid push subcutaneous immunoglobulin replacement therapy during coronavirus disease 2019 shielding
Source: Curr Opin Allergy Clin Immunol. 2022 Sep 27;22(6):371–9. doi: 10.1097/ACI.0000000000000864 (PMC9612677; doi:10.1097/ACI.0000000000000864)
Supplement: Supplemental Digital Content [file coaci-22-371-s006.docx]

Supplementary Table 1. Patients’ experience of hospital based IVIg.

| **What was the main reason you had chosen IVIg in hospital rather than SCIg at home prior to March 2020? (n = 22)** | | |
| --- | --- | --- |
|  |  | n (%) |
|  | I was not offered SCIg at home before | 4 (18) |
|  | I did not want to have to inject myself and administer infusions at home | 8 (36) |
|  | I have tried SCIg previously and my preference was to change back to IVIg | 1 (5) |
|  | IVIg infusions occur less frequently than home SCIg infusions | 2 (9) |
|  | I prefer coming into hospital for Ig treatment | 5 (23) |
|  | Other (please specify) *Never has SCIG before. *Have SCIg in hospital as I prefer that to doing it at home. | 2 (9) |
|  |  | |
| **How long were you receiving IVIg in hospital prior to March 2020? (n = 22)** | | |
|  |  | n (%) |
|  | I had not started receiving IVIg in hospital. | 1 (5) |
|  | Less than 1 year | 1 (5) |
|  | 1-2 years | 5 (23) |
|  | 3-5 years | 4 (18) |
|  | 6-10 years | 4 (18) |
|  | Over 10 years | 7 (32) |
|  |  | |
| **How satisfied were you with your IVIg infusions in hospital prior to March 2020? (Place a X on the scale below) 0% = Extremely unsatisfied, 100% = Extremely satisfied) (n = 22)** | | |
|  |  | n (%) |
|  | 80 - 89% | 2 (9) |
|  | 90 - 99% | 8 (36) |
|  | 100% | 12 (55) |

*IVIg, intravenous immunoglobulin; SCIG, subcutaneous immunoglobulin.*
